# Saturated high‐fat diet‐induced obesity increases adenylate cyclase of myocardial *β*‐adrenergic system and does not compromise cardiac function

**DOI:** 10.14814/phy2.12914

**Published:** 2016-08-31

**Authors:** Danielle F. Vileigas, Adriana F. de Deus, Danielle C. T. da Silva, Loreta C. de Tomasi, Dijon H. S. de Campos, Caroline S. Adorni, Scarlet M. de Oliveira, Paula G. Sant'Ana, Katashi Okoshi, Carlos R. Padovani, Antonio C. Cicogna

**Affiliations:** ^1^ Department of Internal Medicine Medical School São Paulo State University “Júlio de Mesquita Filho” Botucatu São Paulo Brazil; ^2^ Department of Biostatistics Biosciences Institute São Paulo State University “Júlio de Mesquita Filho” Botucatu São Paulo Brazil

**Keywords:** Cardiac remodeling, obesity, saturated high‐fat diet, *β*‐adrenergic pathway

## Abstract

Obesity is a worldwide pandemic associated with high incidence of cardiovascular disease. The mechanisms by which the obesity leads cardiac dysfunction are not fully elucidated and few studies have evaluated the relationship between obesity and proteins involved in myocardial *β*‐adrenergic (*β*A) system. The purpose of this study was to evaluate the cardiac function and *β*A pathway components in myocardium of obese rats. Male *Wistar* rats were distributed into two groups: control (*n* = 17; standard diet) and obese (*n* = 17; saturated high‐fat diet) fed for 33 weeks. Nutritional profile and comorbidities were assessed. Cardiac structure and function was evaluated by macroscopic postmortem, echocardiographic and isolated papillary muscle analyzes. Myocardial protein expression of *β*_1_‐ and *β*_2_‐adrenergic receptors, G*α*s protein, adenylate cyclase (AC) and protein kinase A (PKA) was performed by Western blot. Cardiac cyclic adenosine monophosphate (cAMP) levels and PKA activity were assessed by ELISA. Obese rats showed increased adiposity index (*P* < 0.001) and several comorbidities as hypertension, glucose intolerance, insulin resistance, and dyslipidemia compared with control rats. Echocardiographic assessment revealed increased left atrium diameter (C: 4.98 ± 0.38 vs. Ob: 5.47 ± 0.53, *P* = 0.024) and posterior wall shortening velocity (C: 37.1 ± 3.6 vs. Ob: 41.8 ± 3.8, *P* = 0.007) in obese group. Papillary muscle evaluation indicated that baseline data and myocardial responsiveness to isoproterenol stimulation were similar between the groups. Protein expression of myocardial AC was higher in obese group than in the control (C: 1.00 ± 0.21 vs. Ob: 1.25 ± 0.10, *P* = 0.025), whereas the other components were unchanged. These results suggest that saturated high‐fat diet‐induced obesity was not effective in triggering cardiac dysfunction and impair the beta‐adrenergic signaling.

## Introduction

Obesity is a metabolic chronic disease characterized by excessive fat accumulation in relation to lean tissue mass (World Health Organization, 2016). It is considered a global epidemic and its incidence and prevalence increased significantly in recent decades (World Health Organization, 2000) resulting in more than 1.9 billion people over age 18 with overweight, wherein more than 30% of this population is obese (World Health Organization, 2016). The excess of adipose tissue has been consolidated itself as a nutritional disorder associated with a high incidence of comorbidities, which are related to reduced life expectancy and increased risk of mortality, representing serious public health problem (Olshansky et al. 2005; Abdullah et al. 2011).

Although the association between excess adipose tissue and deleterious effect on the heart has been wide documented (Abel et al. 2008; Aurigemma et al. 2013; Zeng et al. 2015), experimental studies with rodents have shown controversial results for the appearance of cardiac dysfunction in models of obesity induced by high‐fat diets (Carroll et al. 2006; Medei et al. 2010), possibly by no standardization of an appropriate model.

The main targets of studies are the mechanistic pathways related to this cardiac function abnormality, since they are not fully elucidated. Several factors have been presented to be responsible for the cardiac modifications in obesity, including the *β*‐adrenergic (*β*A) system (Strassheim et al. 1992; Carroll et al. 1997; Dincer 2011) that plays an important role in regulating cardiac function and is hyperactive in obesity (Saucerman and McCulloch 2006; Lambert et al. 2010). Thus, investigating components of this pathway is relevant for understanding the mechanisms involved in the cardiac functional changes induced by excess body fat so that could contribute to the development of therapeutic strategies in future.

Few studies have addressed the relationship between obesity and myocardial *β*A system, and the findings are controversial in genetic and dietary obesity model, the latter with a predominance of unsaturated fatty acids (Carroll 1999; Carroll et al. 2002; Minhas et al. 2005; Dincer 2011). Considering the lack of information regarding the relation between obesity promoted by saturated high‐fat diet, cardiac function and *β*A pathway, this study aimed to evaluate the heart performance and components of the *β*‐adrenergic system in the myocardium of obese rats induced by saturated high‐fat diet. In brief, we hypothesized that obesity promotes cardiac dysfunction because of changes in components of myocardial *β*‐adrenergic pathway.

## Methods

### Animals and experimental protocol

Sixty‐day‐old male Wistar rats were obtained from the Anilab Laboratory Animal Creation and Trade (Paulínia, SP, Brazil). After 7 days of acclimatization, the rats were randomized into two groups: control (C, *n* = 17) and obese (Ob, *n* = 17), which were fed a standard diet (SD) and saturated high‐fat diet (SHFD), respectively, for 33 weeks. All rats had free access to food and water. Animals were housed in individual cages with controlled temperature (24 ± 2°C), humidity (55 ± 5%) and light (12‐h light/dark cycle).

The experiments and procedures were performed according to the Guide for the Care and Use of Laboratory Animals published by the National Research Council (National Research Council, 2011) and approved by the Ethics Committee on Animal Experiments of the Botucatu Medical School (protocol number 993/2012).

### Diets

The experimental diets were developed in Experimental Research Unit (UNIPEX) of Botucatu Medical School in partnership with Biotron Zootecnica^®^ (Rio Claro, SP, Brazil), based on a dietary model previously used by the group (Nascimento et al. 2008). The following ingredients were used for formulating diets: corn bran; soybean hulls, and bran; dextrin; salt; vitamin and mineral complex; palm kernel oil and soybean oil. The SD contained 31.0% of its kcal from protein, 51.6% from carbohydrates, and 17.4% from fat; and SHFD, 18.7% from proteins, 41.6% from carbohydrates, and 39.7% from fat. The SHFD was calorically richer (SHFD = 3.85 kcal/g vs. SD = 3.10 kcal/g) because of higher energy from the fat. The content of saturated/unsaturated fatty acids was 61.5/38.5% in SD and 64.8/35.2% in SHFD.

### Nutritional profile

The nutritional profile was evaluated according to the following parameters: food and caloric intake, feed efficiency, weight and fat body, and adiposity index. Animals food and body weight were measured weekly. Caloric consumption was determined by multiplying the energy value of each diet (g × kcal) to the weekly food intake. To analyze the animal's capacity to convert consumed food energy in body weight, feed efficiency was calculated dividing the total body weight gain (g) by total energy intake (Kcal). The adipose tissue fat pads were dissected and weighed after animals had been anesthetized (50 mg/kg ketamine; 1 mg/kg xylazine; i.p.) and decapitated; the amount of total body fat was determined by the sum of epididymal, retroperitoneal, and visceral fat pads. The adiposity index was calculated by total body fat divided by the final body weight and multiplied by 100.

### Obesity‐related comorbidities

#### Systolic blood pressure

The Systolic blood pressure (SBP) was measured in conscious rats using the noninvasive tail‐cuff method with an electro‐sphygmomanometer, Narco Bio‐System (International Biomedical, Austin, TX) at the conclusion of the experiment. The animals were warmed in a wooden box between 38 and 40°C with heat generated by two incandescent lamps for 4 min to cause vasodilation artery tail and were then transferred to an iron cylindrical support that was specially designed to allow total exposure of the animal's tail. After that, a sensor was placed in the proximal region of the tail, coupled to the electro‐sphygmomanometer (Santos et al. 2014). The arterial pulsations were recorded in computerized data acquisition system (AcqKnowledge ® MP100, Biopac Systems Inc., Santa Barbara, CA). The average of two readings was recorded for each measurement.

#### Oral glucose tolerance test and homeostatic model assessment of insulin resistance

At the end of treatment, the animals were fasted for 6 h. Blood samples from the tail tip were collected at baseline and after intraperitoneal administration of 30% glucose solution, equivalent to 2.0 g/kg body weight. Blood samples were collected at 0 min (baseline) and after 15, 30, 60, 90 and 120 min of glucose infusion, and analyzed using a handheld glucometer (Accu‐Chek Go Kit; Roche Diagnostic Brazil Ltda, Sao Paulo, Brazil). Glucose tolerance was assessed by the area under the curve (AUC). The homeostatic model assessment of insulin resistance (HOMA‐IR) was used as an insulin resistance index, calculated according to the formula: HOMA‐IR = [fasting glucose (mmol/L) × fasting insulin (*μ*U/mL)]/22.5 (Matthews et al. 1985).

#### Metabolic and hormonal profile

At the end of experimental protocol, the animals were fasted for 12 h, anesthetized (50 mg/kg ketamine; 1 mg/kg xylazine; i.p.), and euthanized by decapitation. Blood samples were collected and the serum was separated by centrifugation at 1620 *g* for 10 min at 4°C and stored at −80°C for later analysis. The triacylglycerol, total cholesterol, high‐ (HDL) and low‐density lipoprotein (LDL) concentrations were determined using specific kits (BIOCLIN^®^, Belo Horizanto, MG, Brazil) and analyzed by automated colorimetric enzymatic method (Chemistry Analyzer BS‐200, Mindray Medical International Limited, Shenzhen, China). The nonesterified fatty acids (NEFA) levels were evaluated by colorimetric kit (WAKO Pure Chemical Industries Ltda, Osaka, Japão). The hormone levels of leptin and insulin were performed by enzyme‐linked immunosorbent assay (ELISA) method (EMD Millipore Corporation, Billerica, MA). For the glucose analysis, animals were exposed to fasting and anesthesia, as described above, and blood samples were collected from tail tip; the serum glucose levels were assessed using a handheld glucometer.

### Cardiac morphological profile post death

The rats were euthanized and after thoracotomy, the heart was removed and dissected. The presence of cardiac remodeling (i.e., presence or absence of hypertrophy) was determined by macroscopic analysis of the following parameters: heart weight (HW), atrium (AT) and left (LV) and right (RV) ventricles weights and their ratio with tibia length.

### Echocardiographic study

Echocardiographic evaluation was performed 1 week prior to euthanasia using a commercially available echocardiograph (General Electric Medical Systems, Vivid S6, Tirat Carmel, Israel) equipped with a 5–11.5 MHz electronic transducer, in according to previous studies (Leopoldo et al. 2010; Gimenes et al. 2015). Briefly, rats were anesthetized via intraperitoneal injection of a mixture of ketamine (50 mg/kg) and xylazine (1 mg/kg). A two‐dimensional parasternal short‐axis view of the left ventricle (LV) was obtained at the level of the papillary muscles. M‐mode tracings were obtained from short‐axis views of the LV at or just below the tip of the mitral‐valve leaflets, and at the level of the aortic valve and left atrium. M‐mode images of the LV were printed on a black‐and‐white thermal printer (Sony UP‐890MD) at a sweep speed of 100 mm/sec. All cardiac structures were manually measured with a caliper by the same observer according to the method of the American Society of Echocardiography (Lang et al. 2005). Measurements were recorded as the mean of at least five consecutive cardiac cycles.

The following LV structural parameters were evaluated: LV diastolic diameter (LVDD), relative wall thickness (RWT), left atrial (LA), and aortic (AO) diameter. LV function was assessed based on the heart rate (HR), endocardial fractional shortening (FS), posterior wall shortening velocity (PWSV), early and late diastolic mitral inflow velocities (E and A waves), and E/A ratio.

### Isolated papillary muscle function

Cardiac contractile performance was evaluated by studying isolated papillary muscle from LV as previously described (Leopoldo et al. 2010, 2011; Ferron et al. 2015). The following mechanical parameters were measured from isometric contraction: maximum developed tension (DT; g/mm^2^), resting tension (RT; g/mm^2^), peak of positive (+dT/dt; g/mm^2^/sec), and negative (−dT/dt; g/mm^2^/sec) tension derivative. The mechanical behavior of papillary muscle was assessed under baseline conditions at 2.5 mmol/L Ca^2+^ and during *β*‐adrenergic stimulation with 10^−8^, 10^−7^ and 10^−6^ mol/L isoproterenol at 1.0 mmol/L Ca^2+^. All variables were normalized per cross‐sectional area of papillary muscle (CSA).

### Western Blot analysis

Protein expression of the following components of myocardial *β*‐adrenergic system were evaluated: *β*_1_‐ and *β*_2_‐adrenergic receptors (*β*_1_‐AR and *β*_2_‐AR, respectively), stimulatory G protein alpha‐subunit (G*α*s), adenylate cyclase (AC) 5/6 isoforms and protein kinase A (PKA). Briefly, LV samples were rapidly frozen in liquid nitrogen and subsequently homogenized in a solution containing RIPA buffer (Amresco LLC, Solon, OH) and protease (Sigma‐Aldrich, St. Louis, MO) and phosphatase (Roche Diagnostics, Indianapolis, IN) inhibitors. The samples were subjected to SDS‐PAGE in 10% polyacrylamide gel, and thereafter, were electrotransferred to the nitrocellulose membrane (Armsham Biosciences, Piscataway, NJ). The blotted membrane was blocked (5% nonfat dry milk, 20 mmol/L Tris‐HCl pH 7.4, 137 mmol/L NaCl and 1% Tween 20) for 2 h at room temperature and then incubated overnight at 4–8°C with primary antibody against *β*_1_‐AR (1:1000; Abcam, Cambridge, MA), *β*_2_‐AR (1:500; Abcam), G*α*s protein (1:500; Abcam), AC 5/6 isoforms (1:300; Novus Biologicals, Littleton, CO), and PKA (1:200; Abcam). The immunoblots were washed three times with TBS‐T and incubated for 1.5 h with peroxidase‐conjugated anti‐rabbit secondary antibody (1:5000–1:10000; Abcam), then washed three times again with TBS‐T and incubated with ECL (Enhanced Chemi‐Luminescence, Amersham Biosciences, Piscataway, NJ) for chemiluminescence detection. Blots were analyzed on Scion Image software (Scion Corporation, Frederick, MD) and protein expressions were normalized to *β*‐actina expression. (1:1000; Cell Signaling Technology, Danvers, MA).

### Elisa analysis

The concentration of cyclic adenosine monophosphate (cAMP) and PKA activity of LV fragments were determined using ELISA kits (Enzo Life Sciences Ltd, Farmingdale, NY) according to manufacturer's instructions. Each sample was analyzed in triplicate. Absorbance was recorded using an ELISA plate reader (Synergy/HT, Biotek, Winooski, VT).

### Statistical analysis

All results are presented as mean ± standard deviation (SD) and were subjected to Student's *t*‐test for independent samples. In exception, the papillary muscle function after inotropic intervention were evaluated by analysis of variance (ANOVA) on the model of repeated measures for independent groups and complemented by the Bonferroni post hoc test for multiple comparisons when significant differences were found (*P* < 0.05). The level of significance considered was 5% (*α *= 0.05).

## Results

### Nutritional profile and comorbidities

The nutritional profile of the groups is shown in Table 1. Prolonged exposure to SHFD caused a significant increase in final body weight, total body fat and adiposity index. While the body showed a slight increase of 16%, the total body fat and adiposity index increased 127% and 100%, respectively, in the Ob group compared to C. During the experimental period, animals of the Ob group ate less food than those in the C group, however, feed efficiency was higher in Ob animals, and there was no statistical difference in caloric consumption between groups.

**Table 1:** Nutritional profile of animals

Variables	Control (*n* = 17)	Obese (*n* = 17)	*P*
Initial body weight, g	167 ± 15	169 ± 15	0.745
Final body weight, g	469 ± 53	545 ± 66	<0.001
Total body fat, g	20.2 ± 8.3	45.8 ± 10.8	<0.001
Adiposity index, %	4.40 ± 1.4	8.81 ± 1.0	<0.001
Food intake, g	24.3 ± 2.1	19.6 ± 1.9	<0.001
Caloric consumption, kcal	75.2 ± 6.6	75.5 ± 7.3	0.925
Feed efficiency, %	1.31 ± 0.16	1.61 ± 0.18	<0.001

Values are means ± SD. Student's *t*‐test for independent samples.

The comorbidities associated with obesity are summarized in Table 2. Long‐term SHFD‐induced obesity led to significant cardiovascular, metabolic and hormonal changes. The SBP, AUC, HOMA‐IR, insulin and leptin hormones and serum levels of glucose, triacylglycerol, total cholesterol, LDL, and NEFA were higher in Ob rats than in C rats. The HDL levels were similar between groups.

**Table 2:** Obesity‐related comorbidities

Variables	Control (*n* = 11)	Obese (*n* = 11)	*P*
SBP, mmHg^a^	122 ± 10	129 ± 6	0.013
Glucose, mg/dL	104 ± 12	117 ± 14	0.027
AUC, mg dL^−1^ min	18,096 ± 3874	23,845 ± 4535	<0.001
HOMA‐IR	28.7 ± 9.9	46.4 ± 19.0	0.013
Insulin, ng/mL	4.4 ± 1.3	6.5 ± 2.2	0.012
Leptin, ng/mL	3.2 ± 1.5	16.9 ± 5.9	<0.001
Triacylglycerol, mg/dL	41.9 ± 14.2	70.6 ± 28.4	0.007
Total cholesterol, mg/dL	64.8 ± 7.8	77.7 ± 14.0	0.015
HDL, mg/dL	24.5 ± 4.4	27.0 ± 4.9	0.208
LDL, mg/dL	23.1 ± 3.0	27.6 ± 5.5	0.025
NEFA, mmol/L	0.42 ± 0.09	0.53 ± 0.12	0.025

Values are means ± SD. Student's *t*‐test for independent samples.

SBP, systolic blood pressure; AUC, area under the curve for glucose; HOMA‐IR, homeostasis model assessment of insulin resistance; HDL, high‐density lipoprotein; LDL, low‐density lipoprotein; NEFA, nonesterified fatty acids.

a *n* = 17 for control and obese groups.

### Post mortem cardiac morphology

Table 3 shows the influence of obesity on cardiac macroscopic structure in C and Ob rats. There was no significant difference among all studied parameters between the groups (*P > *0.05).

**Table 3:** Postmortem cardiac morphology

Variables	Control (*n* = 17)	Obese (*n* = 17)	*P*
Tibia, cm	4.23 ± 0.12	4.31 ± 0.19	0.167
Heart/Tibia, g/cm	0.251 ± 0.03	0.254 ± 0.02	0.720
Left ventricle/Tibia, g/cm	0.184 ± 0.023	0.184 ± 0.015	0.944
Right ventricle/Tibia, g/cm	0.048 ± 0.007	0.048 ± 0.005	0.791
Atrium/Tibia, g/cm	0.019 ± 0.003	0.021 ± 0.004	0.111

Values are means ± SD. Student's *t*‐test for independent samples.

### Assessment of cardiac structure and function

**Figure 1:**
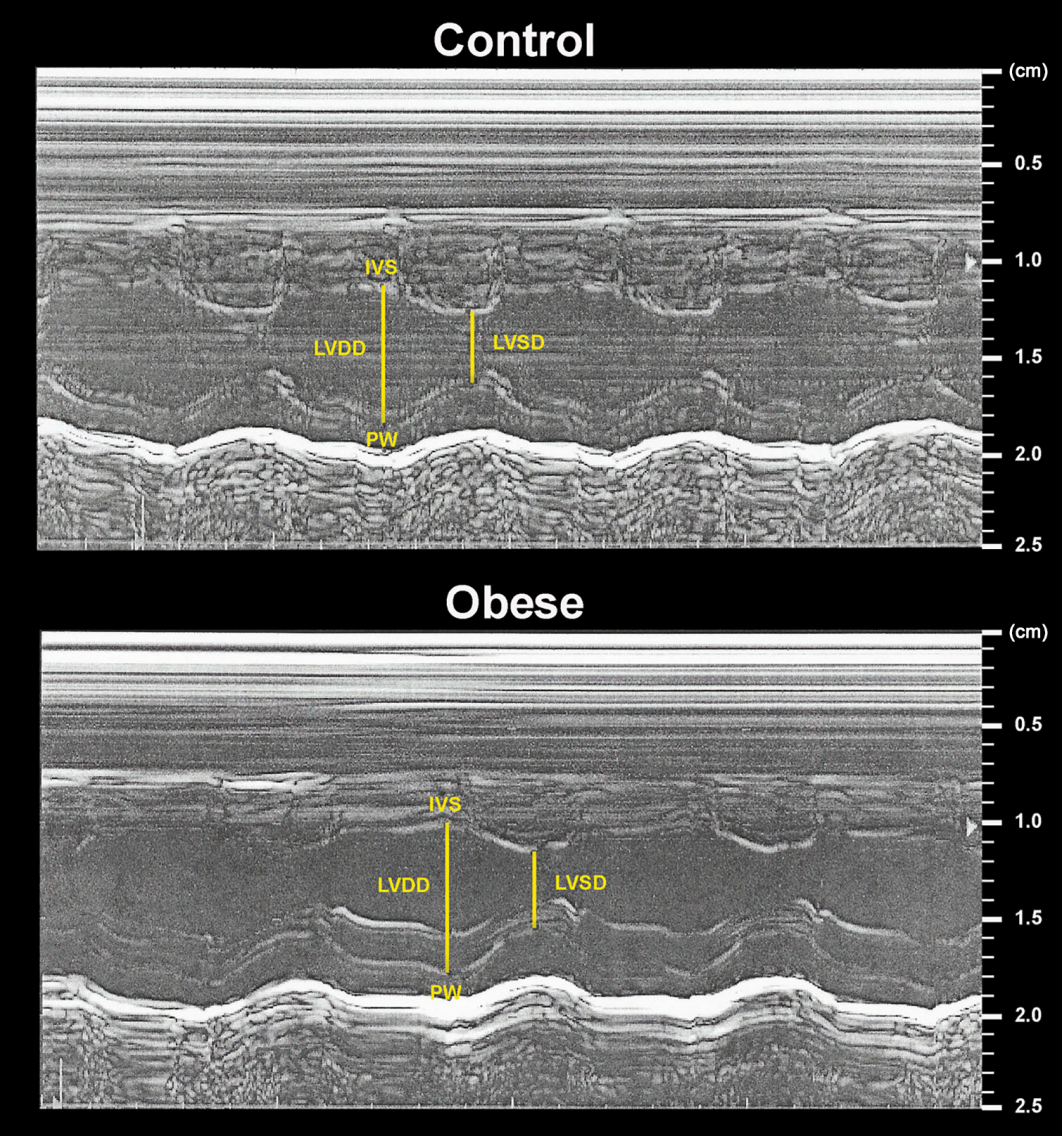
Left ventricle M‐mode echocardiograms from control and obese rats. LVDD and LVSD, left ventricular (LV) diastolic and systolic diameters, respectively; IVS, interventricular septum; PW, posterior wall.

Illustrative LV M‐mode echocardiograms are shown in Figure 1. Structural and functional echocardiographic data are presented in Table 4. LA and LA/AO were significantly greater in Ob group; there were no differences in other morphological variables. All functional parameters did not differ between the groups, except the PWSV, which was higher in the obese rats, suggesting an improvement in systolic function promoted by obesity.

**Table 4:** Echocardiographic assessment

Parameters	Control (*n* = 11)	Obese (*n* = 11)	*P*
Heart rate, bpm	258 ± 47	237 ± 23	0.193
LVDD, mm	7.52 ± 0.27	7.75 ± 0.46	0.159
RWT	0.36 ± 0.02	0.37 ± 0.04	0.549
LA, mm	4.98 ± 0.38	5.47 ± 0.53	0.024
LA/AO	1.27 ± 0.09	1.37 ± 0.11	0.024
FS, %	52.2 ± 4.3	53.9 ± 5.2	0.425
PWSV, mm/sec	37.1 ± 3.6	41.8 ± 3.8	0.007
E wave, cm/sec	67.3 ± 7.0	71.2 ± 7.1	0.210
E/A	1.64 ± 0.25	1.68 ± 0.25	0.716

Values are means ± SD. Student's *t*‐test for independent samples.

LVDD, left ventricle diastolic diameter; RWT, relative wall thickness; LA, left atrial diameter; AO, aortic diameter; FS, endocardial fractional shortening; PWSV, posterior wall shortening velocity; E, early diastolic mitral inflow velocity; A, late diastolic mitral inflow velocity.

**Figure 2:**
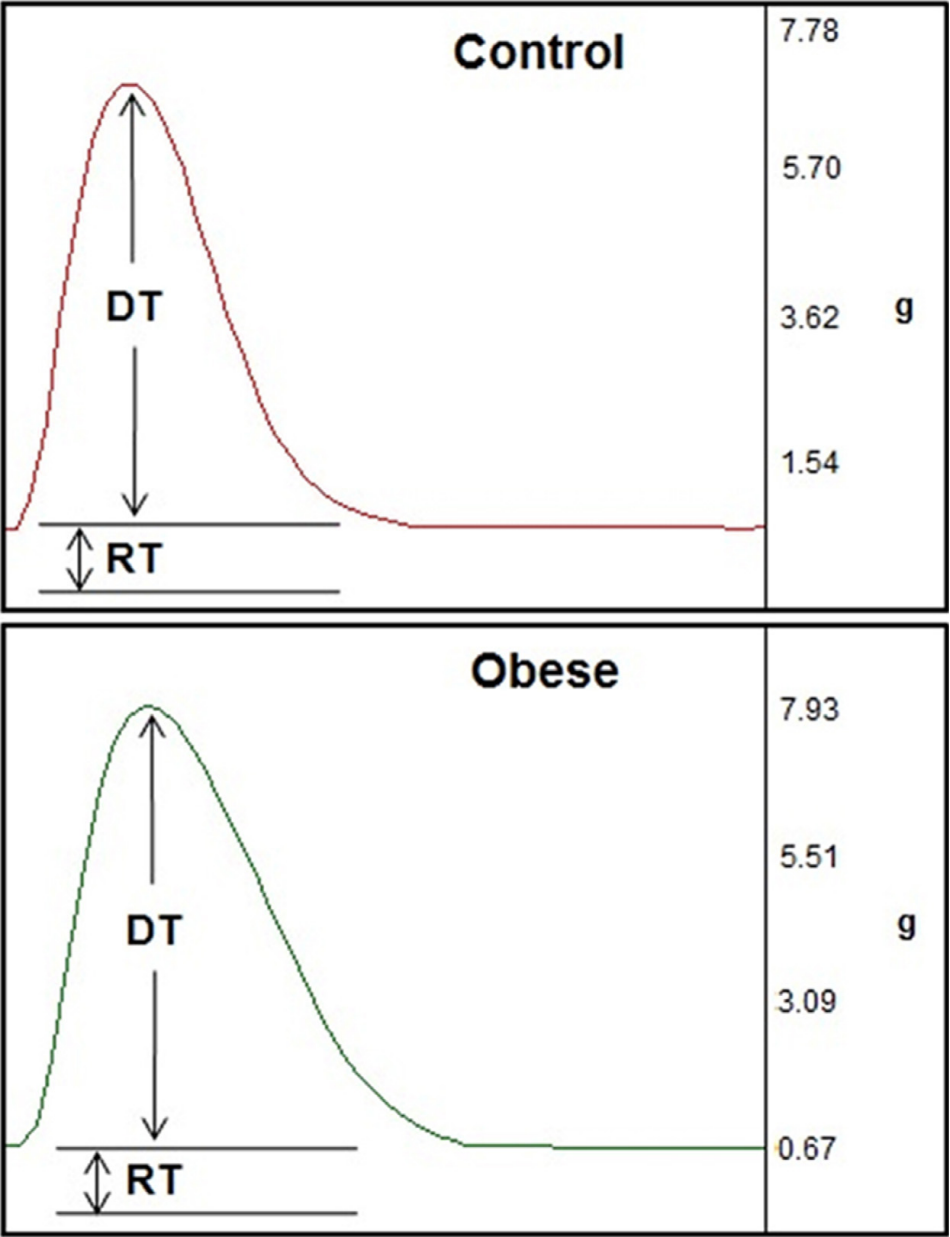
Papillary muscle recordings during isometric contractions at extracellular calcium concentration of 2.5 mmol/L from control and obese rats. DT, maximum developed tension (g); RT, resting tension (g).

**Figure 3:**
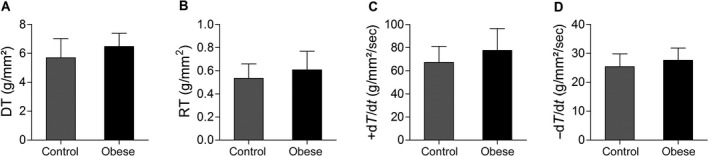
Baseline condition in papillary muscles from control (*n* = 16) and obese (*n* = 15) rats. (A) DT, maximum developed tension (g/mm^2^); (B) RT, resting tension (g/mm^2^); (C) +dT/dt, peak of positive tension derivatives (g/mm^2^/sec); (D) −dT/dt, peak of negative tension derivatives (g/mm^2^/sec). Values are means ± SD. Student's *t*‐test for independents sample.

**Figure 4:**
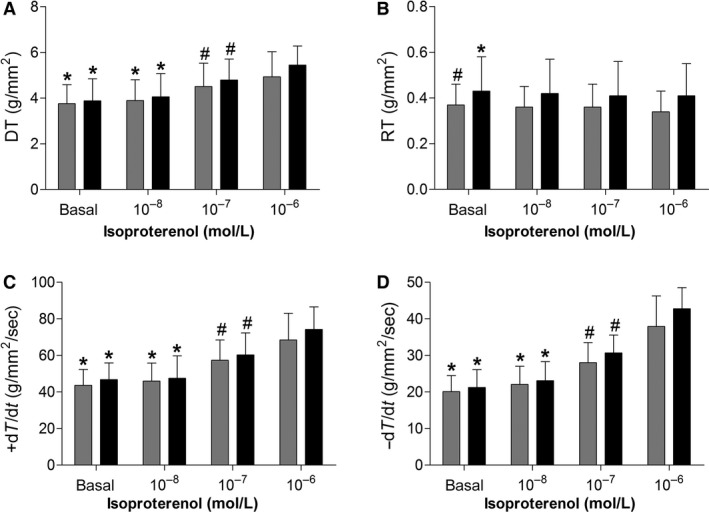
Effects of isoproterenol stimulation on myocardial function in papillary muscles from control (gray bars; *n* = 16) and obese (black bars; *n* = 15) rats. (A) DT, maximum developed tension (g/mm^2^); (B) RT, resting tension (g/mm^2^); (C) +dT/dt, peak of positive tension derivatives (g/mm^2^/sec); (D) −dT/dt, peak of negative tension derivatives (g/mm^2^/sec). Values are means ± SD. *P < 0.05 versus 10–7 and 10–6; ^#^ *P *< 0.05 versus 10–6. ANOVA and Bonferroni.

Illustrative papillary muscle recordings during isometric contractions at extracellular calcium concentration of 2.5 mmol/L are shown in Figure 2. The Figures 3 and 4 summarize papillary muscle functional data from control and obese rats at baseline and after isoproterenol stimulation. The papillary muscle CSA showed no difference between the groups (C, 1.15 ± 0.21 vs. Ob, 1.12 ± 0.24; *P *=* *0.676). Obesity did not lead to mechanic cardiac dysfunction under baseline conditions and after inotropic intervention. Instead, DT and +dT/dt exhibited a trend toward to be significantly higher in the obese animals compared to the control group at baseline (DT: C, 5.7 ± 1.3 vs. Ob, 6.5 ± 0.9, *P *=* *0.082; +dT/dt: C, 67.2 ± 13.8 vs. Ob, 77.6 ± 18.9, *P *=* *0.098; Fig. 3). Although the maneuver has been effective in promoting functional change intragroup for all parameters analyzed, the comparison between the C and Ob groups did not exhibit statistical differences in response to *β*‐adrenoceptor stimulation (Fig. 4).

### Evaluation of beta‐adrenergic system components

**Figure 5:**
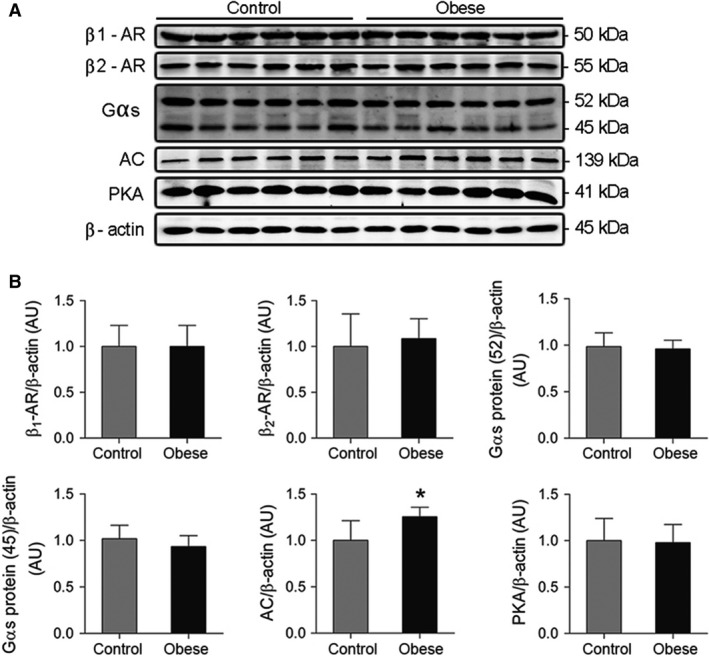
(A) Representative western blots and (B) quantification of myocardial *β*1‐ and *β*2‐adrenergic receptors, Gαs protein (52 and 45 kDa), adenylate cyclase (AC) and rotein kinase A (PKA) from control and obese rats (*n* = 6 in each group). Western blot bands were normalized to *β*‐actin. Values are means ± SD. *P < 0.05 versus control. Student's *t*‐test for independent samples.

The protein expression of the myocardial *β*‐adrenergic system components is presented in Figure 5. Obesity did not alter the myocardial protein levels of *β*_1_‐AR, *β*_2_‐AR, G*α*s protein (52 kDa and 45 kDa) and PKA (*P *>* *0.05). In addition, as shown in Figure 5B, the protein expression of AC was significantly higher in Ob group (C, 1.00 ± 0.21 vs. Ob, 1.25 ± 0.10; *P *=* *0.025).

**Figure 6:**
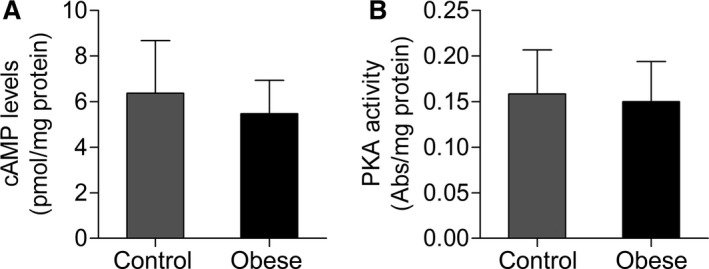
(A) Cyclic adenosine monophosphate (cAMP) levels and (B) protein kinase A (PKA) activity in cardiac tissue from control and obese rats (*n* = 10 in each group). Values are means ± SD. Student's *t*‐test for independent samples.

Obesity promoted no change in the cAMP levels and PKA activity in cardiac tissue (*P *>* *0.05), as showed in Figure 6.

## Discussion

The aim of this study was to investigate whether the obesity leads to cardiac dysfunction and if this is associated to changes, at the molecular level, of the mains cardiac *β*A system components. The major finding was that excess adipose tissue did not cause cardiac dysfunction and there was subtle increase in protein expression of AC without compromising other mediators involved in myocardial *β*A pathway evaluated in this study.

Obesity models induced by high‐calorie diet have become an important tool for studying the pathophysiological effects in different organs. The long period treatment with high‐fat diet used in this study was effective to promote excess adipose tissue in rats, in agreement with other authors (Buettner et al. 2006; Leopoldo et al. 2010; Liu et al. 2016). Furthermore, the obesity led to several comorbidities such as hypertension, dyslipidemia, glucose intolerance, insulin resistance, hyperinsulinemia, and hyperleptinemia; these findings are consistent with other researches (Dobrian et al. 2000; Huang et al. 2004; Higa et al. 2014) and compatible with metabolic syndrome signals. Therefore, the results of the nutritional profile and comorbidities, taken together, constitute a representative and corresponding condition to human obesity.

Cardiac structural morphological post mortem evaluation revealed no alterations, such as the presence of atrial and ventricular hypertrophy, in accordance with certain authors (Carroll et al. 2006; Campos et al. 2014) and in disagreement with other researchers (Relling et al. 2006; Leopoldo et al. 2011; Lima‐Leopoldo et al. 2014). However, echocardiographic structural study showed increased atrial dimension in the obese group that was not accompanied by other anatomical modifications. Studies have presented the obesity may promote LA enlargement and predispose to atrial fibrillation (Wang et al. 2004; Movahed and Saito 2008; Nalliah et al. 2016). One possible explanation for the large LA diameter is the hypervolemic and hyperdynamic circulation found in obesity, which entails elevated filling pressures of the cardiac cavities (Pascual et al. 2003; Bayes‐Genis et al. 2007). As the atrium is a chamber of fine and complacent walls, it would be the first to suffer the consequences of this changed circulatory status.

The LV function evaluation by echocardiography and isolated papillary muscle revealed that the heart performance not deteriorated, presenting data consistent with possible function improvement, in contrast to initial hypothesis; thus, while the PWSP was significantly higher in the obese group, the DT and +dT/dt presented, at baseline condition, tendency toward be higher in these animals. In accordance with our echocardiographic results, Lima‐Leopoldo et al. (2014) observed systolic function improvement in obese rats induced by unsaturated high‐fat diet (UHFD) in the period of 30 weeks; authors suggest that the increase in FS and PWSV may be related to decreased after load and improved LV contractility; in addition, Oliveira Junior et al. (2013) revealed, in obese rats by UHFD during 20 weeks, improved left ventricular systolic performance assessed by FS. In opposition, studies performed in obesity induced by SHFD (Okere et al. 2006; Martins et al. 2015) and UHFD (Carroll et al. 2006; Okere et al. 2006; Medei et al. 2010), which vary from 8 to 20 weeks of treatment, showed no functional changes; however, other authors reported cardiac dysfunction in mice fed with UHFD for 20 weeks (Park et al. 2005; Cao et al. 2016), as well as Relling et al. (2006), using obese rats in 12 weeks of treatment with high‐fat diet. The papillary muscle results of current investigation showed that obesity did not cause dysfunction when it was evaluated at baseline condition and on stimulation by isoproterenol. These data are in agreement with several authors who analyzed the muscle at baseline (Relling et al. 2006; Leopoldo et al. 2010, 2011; Lima‐Leopoldo et al. 2014; Ferron et al. 2015) and under *β*A stimulation (Lima‐Leopoldo et al. 2011; Ferron et al. 2015), and oppose to Carroll et al. (1997), who observed decreased responsiveness to isoproterenol in obese rabbits fed with UHFD for 12 weeks.

Many factors can be pointed as responsible for the differences found in heart function findings in obesity models promoted by intake of high‐fat diets as mentioned above. Among these factors, highlight the following: time of exposure to a high‐fat diet; percentage from the fat regarding to total calories; types of used fatty acids, saturated or unsaturated, as well as their source, animal or vegetable; species and strain of animals; methodologies performed and finally, the presence or absence of comorbidities. These multiple aspects involved in studies that aim to evaluate the influence of obesity on certain organs, makes it difficult to find a uniformity of findings, resulting in different response types. We believe that the diet used in this study may have been one of the causes for the noninduction of cardiac dysfunction, either by content (39.7% kcal from fat) or chosen fat source (particularly palm kernel oil). Recent studies have used high‐fat and high‐sucrose diets to mimic the functional cardiac changes resulting from obesity (Panchal et al. 2012; Carbone et al. 2015; Gonçalves et al. 2016; Sverdlov et al. 2016); these diets have been termed Western diet, which reproduce more accurately the contemporary food consumption (Brainard et al. 2013; Zeeni et al. 2015). The standardization of dietary obesity models which result in cardiac dysfunction is essential for conducting mechanistic studies; however, often the authors did not adequately provide the nutritional information of diets, making them unrepeatable.

The myocardial *β*‐adrenergic pathway is one of the mechanisms that regulate cardiac performance, since it leads to alterations of intracellular Ca^2+^ handling. This pathway is activated by a signaling cascade initiated by the binding of catecholamines or adrenergic agonists to their receptors, followed by interaction with the G*α*s protein, and subsequent activation of AC, which converts ATP to cAMP (Xiao 2001; Lohse et al. 2003). The main target of cAMP is the PKA that phosphorylates several key proteins, modulating the cardiac contraction relaxation (Bers 2002).

The current investigation revealed significant increase in protein expression of AC resulting from obesity, not followed by changes in the other components of *β*A pathway evaluated. Although there are information concerning the factors that modulate AC activity (Sunahara and Taussig 2002), mediators involved in the protein expression regulation remain poorly understood, being suggested leptin as regulator, for acting transcriptional level (Illiano et al. 2002). Since AC leads to the cAMP formation, the increased expression of this enzyme should be accompanied by higher concentration of this nucleotide and consequent PKA activation, which were not observed in this study. Our suggestion is that increased AC would be a compensation to its decreased catalytic activity. Thus, Illiano et al. (2002) evaluated the acute and chronic effects of leptin on the AC activity in heart cells; leptin caused a reduction in the activity of this enzyme after long‐term exposure to this hormone. As in this study, the obesity period and consequent hyperleptinemia were of long duration, it may be hypothesized that this condition should have led a decreased activity of this protein, triggering increased AC concentration as compensatory mechanism. According to the literature, the decremented enzyme activity has been observed in different genetic models of obesity (Chatelain et al. 1981; Strassheim et al. 1992; Hohl et al. 1993). On the other hand, since the AC activity was not measured and, the downstream effectors of AC (i.e., cAMP and PKA) remained unchanged, it can be that subtle increase of 25% in the AC protein expression had no effect on beta‐adrenergic signaling and consequently on the heart function.

As reported previously, excess adipose tissue did not affect the other *β*A pathway components; these results are in agreement with some authors and at odds to others. In a recent study, Ferron et al. (2015) showed that the myocardial protein expression of *β*_1_‐AR, *β*_2_‐AR and G*α*s protein were not altered in obese rats induced by UHFD for 15 weeks; the authors concluded that cardiac damage in the relaxation phase found these animals is not associated with defects in the *β*A system but rather to changes in intracellular Ca^2+^ handling. Other authors also did not identify modifications in cardiac *β*_1_‐AR and *β*_2_‐AR from ob/ob mice and obese rabbits by fat‐enriched diet (Carroll et al. 2002; Minhas et al. 2005). In contrast, Dincer (2011), assessing obese Ossabaw pigs induced by high‐fat diet for 50 weeks, found decreased protein expression of *β*‐adrenergic receptors in the heart, due to increased activity of the sympathetic nervous system, resulting in down‐regulation mechanism and/or desensitization receptors. Minhas et al. (2005) evidenced, in ob/ob mice, a decrease in G*α*s protein expression and PKA activity, which have been restored after exogenous administration of leptin, suggesting the participation of deficiency of this hormone. Different authors have observed that the protein expression and activity of PKA did not change in dietary obesity model (Moberly et al. 2013; Freire et al. 2014), corroborating our data. Overall, the discrepancy in these findings seems to be associated with the use of genetic models of obesity, exposure time to high‐fat diet or, even with the dietary composition.

In conclusion, the obesity induced by saturated high‐fat diet was not effective in triggering cardiac dysfunction and impair the beta‐adrenergic signaling. Further studies are required to evaluate the *β*A pathway components by different methods and to test other diets types, so that adequate study model may be standardized and reproducible in future researches.

## Conflict of Interest

None of the authors has any kind of conflict of interest, financial or otherwise related to this work.
